# Studies on the catalytic domains of multiple JmjC oxygenases using peptide substrates

**DOI:** 10.4161/15592294.2014.983381

**Published:** 2015-01-27

**Authors:** Sophie T Williams, Louise J Walport, Richard J Hopkinson, Sarah K Madden, Rasheduzzaman Chowdhury, Christopher J Schofield, Akane Kawamura

**Affiliations:** 1 Chemistry Research Laboratory; Oxford, UK; 2 Radcliffe Department of Medicine; Division of Cardiovascular Medicine; Wellcome Trust Center for Human Genetics; Oxford, UK

**Keywords:** demethylation, Epigenetics, histone, methyllysine, JmjC histone demethylase, 2OG oxygenases

## Abstract

The JmjC-domain-containing 2-oxoglutarate-dependent oxygenases catalyze protein hydroxylation and *N^ε^*-methyllysine demethylation via hydroxylation. A subgroup of this family, the JmjC lysine demethylases (JmjC KDMs) are involved in histone modifications at multiple sites. There are conflicting reports as to the substrate selectivity of some JmjC oxygenases with respect to KDM activities. In this study, a panel of modified histone H3 peptides was tested for demethylation against 15 human JmjC-domain-containing proteins. The results largely confirmed known *N^ε^*-methyllysine substrates. However, the purified KDM4 catalytic domains showed greater substrate promiscuity than previously reported (i.e., KDM4A was observed to catalyze demethylation at H3K27 as well as H3K9/K36). Crystallographic analyses revealed that the *N^ε^-*methyllysine of an H3K27me3 peptide binds similarly to *N^ε^-*methyllysines of H3K9me3/H3K36me3 with KDM4A. A subgroup of JmjC proteins known to catalyze hydroxylation did not display demethylation activity. Overall, the results reveal that the catalytic domains of the KDM4 enzymes may be less selective than previously identified. They also draw a distinction between the *N^ε^*-methyllysine demethylation and hydroxylation activities within the JmjC subfamily. These results will be of use to those working on functional studies of the JmjC enzymes.

## Abbreviations and acronyms

2OG: 2-oxoglutarate
FIH: Factor Inhibiting HIF
H3: histone 3
HIF: Hypoxia Inducible Factor
JmjC: Jumonji *C*-terminal
JmjN: Jumonji *N*-terminal
KDM: Lysine Demethylase
LSD: Lysine Specific Demethylase
MALDI-TOF MS: Matrix Assisted Laser Desorption/Ionization Time of Flight Mass Spectrometry
MINA53: Myc-Induced Nuclear Antigen with a molecular mass of 53 kDa
NO66: Nucleolar protein 66
PHD: Plant Homeodomain
Rp: Ribosomal protein
TPR: Tetratricopeptide repeat

## Introduction

Post-oligomerization modifications to the nucleic acid and protein components of chromatin play central roles in transcriptional regulation. While DNA methylation is associated with gene silencing, methylation of histone proteins can be activating or repressive depending on the context of the modification.^1^ The introduced methyl groups can undergo further modification, often by oxidation; the largest identified class of such enzymes are the Fe(II)- and 2-oxoglutarate- (2OG) dependent oxygenases, which catalyze oxidation of 5-methylcytosine bases, and of *N*-methyl groups in DNA (and RNA) and histone proteins.^2^

**Figure 1.**
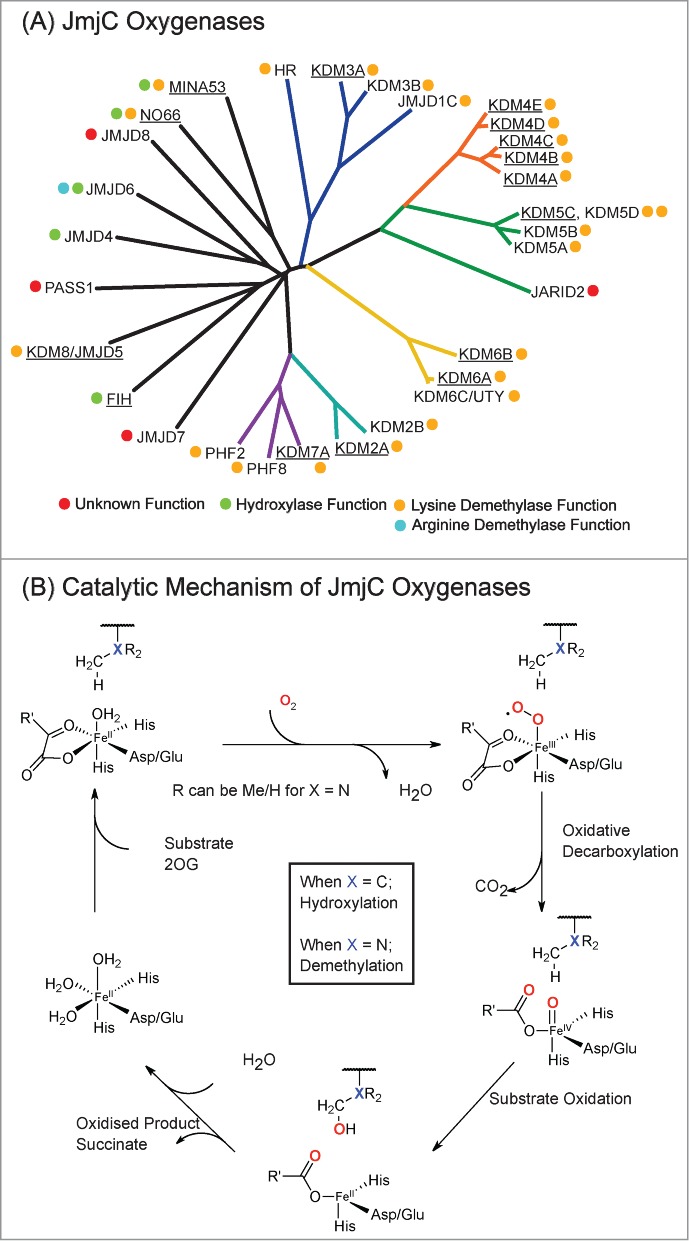
**JmjC Oxygenases share sequence homology and catalytic mechanisms.** (**A**) Phylogenetic analysis of the catalytic domains of human JmjC oxygenases. Reported catalytic functions are indicated by colored circles. MINA53, NO66 and JMJD6 have been reported to be demethylases but have subsequently been shown to have hydroxylase activities.^4-6,8,9^ KDM6C (UTY) was recently identified as a histone demethylase *in vitro*, acting on H3 peptide fragments methylated at H3K27.^43^ HR is Hairless Protein, a recently identified H3K9 demethylase.^44^ Enzymes used in this work are underlined. (**B**) Outline mechanism of JmjC oxygenase catalysis. Oxidative decarboxylation of 2-oxoglutarate (2OG) in the active site forms a highly reactive iron(IV)-oxo intermediate, which hydroxylates the substrate. In the case of demethylation (X = N), the hydroxylated product is unstable and fragments to produce the demethylated species and formaldehyde. The exact protonation states of water molecules complexed to the iron(II) species are unknown.

Two families of *N^ε^*-methyllysine demethylases (KDMs) have been identified: the Fe(II)- and 2OG-dependent JmjC KDMs (**Fig. 1A**) and the flavin-dependent lysine specific demethylases (KDM1s or LSDs).^3^ JmjC KDMs act on all 3 *N^ε^*-methyllysine methylation states (**Fig. 1B**), whereas the KDM1s can only act on di- and mono-methylation states. The JmjC KDMs are part of the wider JmjC subfamily of 2OG oxygenases, which also catalyze protein hydroxylation not involving demethylation.^4^ For several JmjC enzymes, (JMJD6, MINA53 and NO66), there are reports of both demethylation and hydroxylation activities.^4-9^ Substrate promiscuity is well-established in 2OG oxygenase biochemistry, including for the JmjC hydroxylase factor inhibiting hypoxia inducible factor (FIH), which catalyzes hydroxylation of an asparaginyl residue in Hypoxia Inducible Factor α (HIF-α) as well as multiple asparagine-, aspartate- and histidine-residues in ankyrin repeat-domain proteins.^10-13^ Given the different reports of the reactions catalyzed by some JmjC oxygenases, we considered that it would be useful to carry out a systematic study on the biochemical selectivity of the catalytic domains of human JmjC KDMs. We appreciate that other factors, for example, ancillary non-catalytic domains, targeting proteins, protein conformation, co-substrate and inhibitor availability, substrate length (peptide/protein), and cellular compartmentalization, may alter hydroxylation and demethylation catalysis and substrate selectivities in a cellular context. However, these results provide an *in vitro* comparative substrate profiling of JmjC oxygenases that will be useful to other researchers in the field.

In general, the results with JmjC KDMs support the reported selectivities,^14-20^ as well as current structural studies,^21^ which show that JmjC KDMs and hydroxylases are different subfamilies within the 2OG oxygenase family, with little overlap in hydroxylation and demethylation activities. However, we observed that the KDM4 subfamily catalytic domains could not only catalyze demethylation of H3 peptides methylated at K9 and K36, but also at K27, implying that the selectivities of the JmjC KDMs may extend beyond the already characterized substrates.

## Results

To compare the selectivities of the catalytic domains of the JmjC oxygenases we screened a set of H3 peptides (either from Alta Bioscience, or synthesized in house) with the isolated catalytic domains of 15 JmjC oxygenases using MALDI-TOF Mass Spectrometry (MS, **Table 1**). The enzymes were chosen to represent different subfamilies of JmjC oxygenases (**Fig. 1A**) and based on the availability of active recombinant proteins.^22^ The H3 fragment peptides screened were 21 residues in length and included mono-, di-, and tri-methyllysines at K4, K9, K27, and K36 sites (**Table S1**). Standard conditions of 1 μM enzyme, 200 μM 2OG, 10 μM Fe(II) and 100 μM ascorbate were used throughout (i.e., large excesses of cofactors and substrates),^23^ but the assay buffer conditions were optimized to enhance activity and MS signal, as well as to reduce protein precipitation (**Table S2**).

**Table 1.** Summary of initial activity screening of JmjC oxygenases and methylated histone H3 fragment peptides. KDM activity (i.e., greater than 10% substrate demethylation as detected by MALDI mass spectrometry) was observed for all tested members of the KDM enzymes (ticks), including at reported methylation sites (green boxes) and, in the case of the KDM4 enzymes, at novel methylation sites (yellow boxes). No demethylation activity within limits of detection was observed for the putative demethylases MINA53, NO66 or JMJD5 (crosses, red boxes; note for JMJD5 construct-dependent demethylation activity is reported by Hsia et al (dashed red box) 7), while hydroxylation of reported non-histone substrates were observed for MINA53 and NO66 For JMJD5, prime substrate uncoupled 2OG turnover was observed indicating active construct (ticks, green boxes). In some cases, demethylation of the me2/1 state (to the me1/0 state respectively) was only observed when the me2/1 peptide was produced as an intermediate during demethylation of the me3/2 state (dashed green/yellow boxes)

		H3K4			H3K9			H3K27			H3K36			
Subfamily	Enzyme	me3	me2	me1	me3	me2	me1	me3	me2	me1	me3	me2	me1	Activity
KDM2	2A_1–517_	χ	χ	χ	χ	χ	χ	χ	χ	χ	χ	✓	✓	
KDM3	3A_515–1317_	χ	χ	χ	χ	✓	✓	χ	χ	χ	χ	χ	χ	
KDM4	4A_1–359_	χ	χ	χ	✓	✓	χ	✓	✓	χ	✓	✓	χ	
	4B_1–365_	χ	χ	χ	✓	✓	χ	✓	χ	χ	✓	✓	χ	
	4C_1–366_	χ	χ	χ	✓	✓	χ	✓	✓	χ	✓	✓	χ	
	4D_1–358_	χ	χ	χ	✓	✓	χ	χ	χ	χ	χ	χ	χ	
	4E_1–337_	χ	χ	χ	✓	✓	✓	✓	✓	χ	χ	χ	χ	
KDM5	5C_1–765_	✓	✓	✓	χ	χ	χ	χ	χ	χ	χ	χ	χ	
KDM6	6A_940–1401_	χ	χ	χ	χ	χ	χ	✓	✓	χ	χ	χ	χ	
	6B_1141–1590_	χ	χ	χ	χ	χ	χ	✓	✓	✓	χ	χ	χ	
KDM7	7A_38–480_	χ	χ	χ	χ	✓	✓	χ	✓	✓	χ	χ	χ	
Hydrox.	MINA53_26–465_	χ	χ	χ	χ	χ	χ	χ	χ	χ	χ	χ	χ	✓
	NO66_116–641_	χ	χ	χ	χ	χ	χ	χ	χ	χ	χ	χ	χ	✓
	JMJD5_1–416_	χ	χ	χ	χ	χ	χ	χ	χ	χ	χ	χ	χ	✓
	FIH_1–348_	χ	χ	χ	χ	χ	χ	χ	χ	χ	χ	χ	χ	✓

The results with the catalytic domains of KDM2A, KDM3A, KDM5C, KDM6A, and KDM6B support literature assignments.^15,16,18,19,24-28^ Thus, under standard conditions, and as summarized in **Table 1**, (i) KDM2A only accepted H3K36me2 and H3K36me1 (**Figure S1**), (ii) KDM3A accepted H3K9me2 and H3K9me1 (**Figure S2**), (iii) KDM5C accepted H3K4me3, H3K4me2, and H3K4me1 (**Figure S3**), and (iv) KDM6B acted on H3K27me3, H3K27me2 and H3K27me1, while KDM6A acted (under our conditions) on H3K27me3 and H3K27me2, but not H3K27me1 (**Figure S4**). The latter result is consistent with previous reports which demonstrate that the tetratricopeptide repeat (TPR) domain of KDM6A is required for effective H3K27me1 demethylation, possibly by promoting enhanced substrate binding.^16^ KDM7A is reported to catalyze the demethylation of di- and mono-methylated forms of both H3K9 and H3K27 in the absence of substrate methylation at H3K4 (H3K4me3), but only at H3K27 when H3K4 is trimethylated (i.e., H3K4 methylation promotes demethylation at H3K27).^29,30^ We observed the same substrate selectivity for peptides not methylated at H3K4 with our construct of KDM7A, which contains both the JmjC and PHD domains (**Figure S5, S6**).

**Figure 2.**
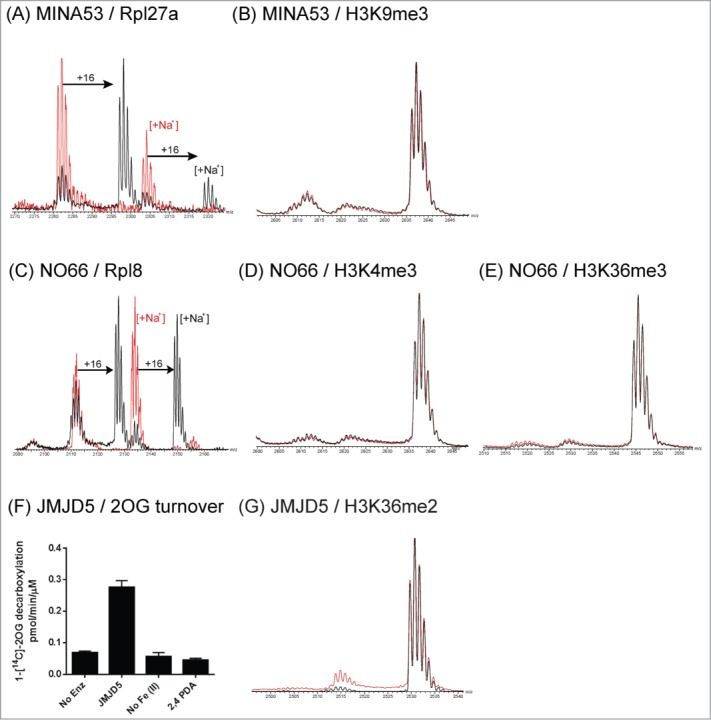
**JmjC Oxygenases MINA53, NO66 and JMJD5 do not catalyze demethylation of histone peptides.** In addition to putative demethylation activities, MINA53 and NO66 have been characterized as hydroxylases acting on ribosomal proteins Rpl27a and Rpl8 respectively. Hydroxylation activities were observed for MINA53 and NO66, acting on Rpl27a and Rpl8 peptide fragments respectively (**A** and **C**); no demethylation was observed with methylated histone peptides (**B**, **D** and **E**). Prime-substrate uncoupled turnover of 2OG by JMJD5 (residues 1–416) was observed in a [^14^C]-labeled 2OG assay, which was dependent on the presence of iron(II) and inhibited by the broad-spectrum 2OG oxygenase inhibitor 2,4-pyridinedicarboxylic acid (2,4 PDA) (**F**). However, demethylation of an H3K36me2 histone peptide was not observed (**G**). Control reactions without added protein are in red.

In the case of the tested JmjC oxygenases assigned as hydroxylases (FIH and the recently reassigned NO66 and MINA53),^4,12,13^ no KDM activity was observed with any of the tested H3 peptides under our assay conditions (**Figs. 2B**, **D, E, S7, S8**). This is of note as NO66 and MINA53 were initially characterized as histone demethylases.^8,9^ Recent work, however, has shown MINA53 and NO66 to be histidyl-hydroxylases acting on the ribosomal proteins Rpl27a and Rpl8, respectively.^31^ In positive controls, we found these reported non-histone peptide substrates to be hydroxylated by MINA53 and NO66 (**Figs. 2A, C**). JMJD5 has also been reported to act as an H3K36me2 demethylase;^7^ however, under our assay conditions, JMJD5-catalyzed demethylation of H3K36me2 peptide was not observed (**Figs. 2F, 2G**, **S10**).

**Figure 3.**
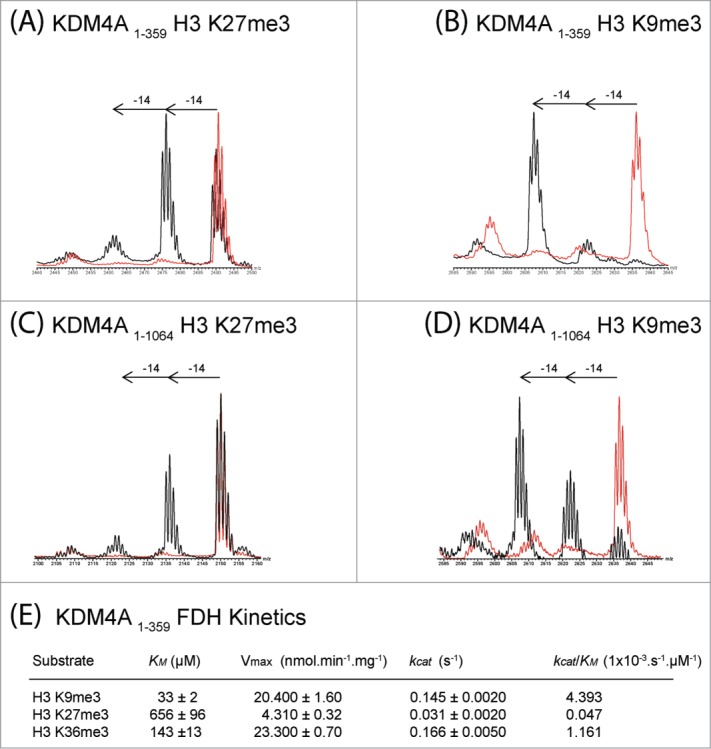
**KDM4A catalyzes demethylation of histone fragment peptides methylated at H3K27.** MALDI-TOF spectra of KDM4A (1–359) catalyzed demethylation of (**A**) H3K27me3 peptide (Biotin-Ahx(aminohexanoic acid)-KAPRKQLATKAAR**Kme3**SAPATGG), (**B**) H3K9me3 peptide (Biotin-Ahx-ARTKQTAR**Kme3**STGGKAPRKQLA). MALDI-TOF spectra of full-length KDM4A (1–1064) catalyzed demethylation of (**C**) H3K27me3 peptide (KAPRKQLATKAAR**Kme3**SAPATGG) and (**D**) H3K9me3 peptide (Biotin-Ahx-ARTKQTAR**Kme3**STGGKAPRKQLA). FLAG-tagged full-length KDM4A (1–1064) was produced in HEK293T cells and purified from cell lysates using anti-FLAG beads prior to reaction with the histone peptide. MALDI-TOF spectra of reactions with (standard conditions, black) and without added enzyme (red) for (**A**)–(**D**) are shown. (**E**) Kinetic parameters determined for KDM4A (1–359) catalyzed demethylation of *N^ε^*-trimethylated H3K9, H3K27 and H3K36 fragment peptides using FDH assay (**Figure S16**).

In the case of the KDM4 subfamily, initial screening results with KDM4A suggested that they may be more promiscuous than reported (**Table 1**, **Figure 3A, B**, **Figure S11**);^14^ hence, we analyzed all 4 human KDM4 subfamily members (KDM4A-D) and the human pseudogene KDM4E (**Table 1**, **Figures S11–15**). As reported, all the KDM4 members acted on H3K9me3 and H3K9me2, although we only observed clear demethylation of H3K9me1 to the H3K9me0 state by KDM4E (although not apparent under the standard conditions, prolonged incubations with KDM4 enzymes leads to demethylation of H3K9me1) (**Figure S15**).^14^ Also, as reported, KDM4A-C catalyzed demethylation of H3K36me3/H3K36me2 but KDM4D/E did not (**Figure S14–S15**).^14^ Surprisingly, most KDM4 catalytic domains also acted on H3K27me3 and in one cases H3K27me2 (**Figures S11–15**).

To investigate the unexpected results with respect to KDM4 selectivity, kinetic analyses were then undertaken using the KDM4A catalytic domain (**Fig. 3E**, **S16**). The H3K9me3 and H3K36me3 modified peptides had similar *k*_cat_ values (*k*_cat_ = 0.145 s^−1^ and 0.166 s^−1^, respectively), but H3K9me3 manifested the highest *k_cat_/K_m_* (0.004393 s^−1^.μM^−1^ for H3K9me3 and 0.001161 s^−1^.μM^−1^ for H3K36me3), consistent with previous reports.^32,33^ The H3K27me3 peptide was a poorer substrate than both the H3K9me3 and H3K36me3 peptides but still displayed substantial activity (*k*_cat_ = 0.031 s^−1^). As anticipated, the *K_m_* value was lowest for H3K9me3 (*K_m_* = 33 μM) followed by H3K36me3 *(K_m_* = 143 μM). Competition experiments, monitoring demethylation of 2 equimolar peptides simultaneously, support the reduced demethylation efficiency of KDM4A toward H3K27me3 relative to H3K9me3/H3K36me3 (**Figure S17**). The majority of H3K9me3 and H3K36me3 peptides were demethylated to the monomethyllysine state before demethylation of H3K27me3 was observed.

**Figure 4.**
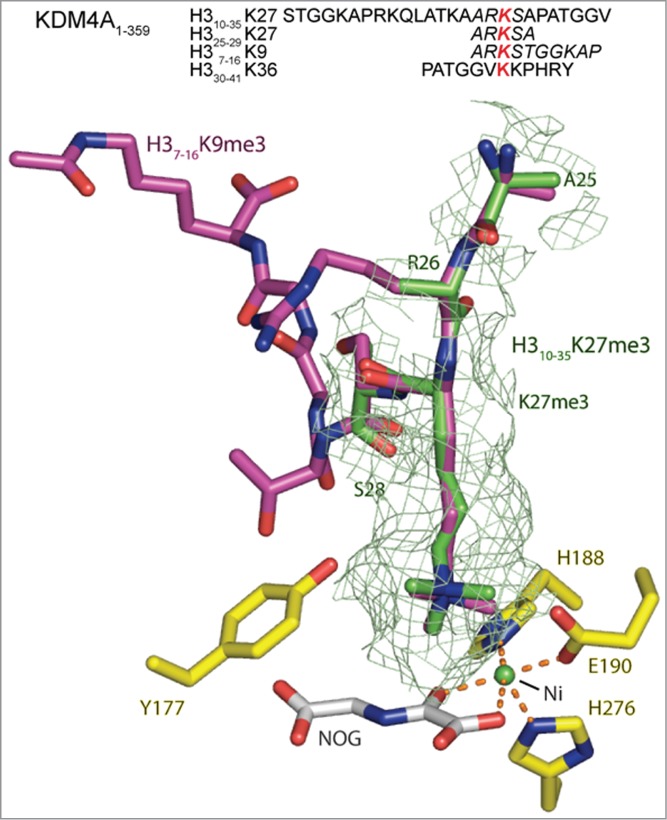
View from an X-ray crystal structure of the catalytic domain of KDM4A in complex with an H3K27me3 fragment peptide (PDB ID: 4V2W). The active site residues are highlighted in yellow. The visible residues of the 25mer H3_10–35_K27me3 (green) peptide is shown overlaid with H3K9me3 (pink), as complexed with KDM4A (PDB ID: 2OQ6, nickel substituted for iron, and *N*-oxalylglycine substituted for 2OG). The position of the H3K27me3 residue of the fragment peptide correlates closely with that of H3K9me3 (and H3K36me3, **Figure S18**). However, in the H3_10–25_K27me3 derived structure only the electron density for the tri-methyl lysine and the residues either side (H3R26 and H3S28) are clearly defined, suggesting the other residues are bound less tightly than the comparable H3K9 and H3K36 substrates. A second structure of a shorter 5 residue H3_25–29_K27me3 peptide in complex with KDM4A (PDB ID: 4V2V) overlays well with that of the 25 residue peptide (**Figure S19**). The sequences for the H3K9, K27 and K36 peptides present in these crystal structures are included with residues for which electron density is observed in italics. The red lysine residue marks the position of the *N^ε^*-trimethylated lysine.

To investigate the mode of KDM4A binding to the H3K27me3 substrate, we solved crystal structures of the KDM4A catalytic domain complexed with both 25 residue and 5 residue H3K27me3 peptides (**Figs. 4**, **S18, S19**). In both cases, the H3K27me3 residue was observed to bind in a near identical manner to both H3K9me3 and H3K36me3 residues in H3 peptides, supporting the proposed activity at H3K27me3 (**Figs. 4**, **S18**). Notably, in the longer 25 residue H3K27me3 peptide complex, density was observed for the trimethyllysine and the 2 adjacent peptide residues (H3R26 and H3S28 respectively), but not for the other residues, while density was observed for all residues of the shorter 5 residue H3K27me3 peptide (**Figure S19**). The lack of observed density for the longer peptide may reflect relatively weaker binding relative of the H3K27me3 compared to the H3K9me3 and H3K36me3 peptides, consistent with the kinetic results (**Figs. 3E**, **S16**).

To investigate whether KDM4A promiscuity is a result of using truncated protein (i.e., constructs containing only the catalytic domain), we used immunoprecipitated FLAG-tagged full-length recombinant KDM4A (which contains JmjN, JmjC, double PHD and tandem tudor domains, **Figure S5**) exogenously over-expressed in HEK 293T cells and tested for demethylation activity. The full-length KDM4A catalyzed demethylation of H3K9me3, K36me3 and K27me3 (**Fig. 3C, D**).

## Discussion

Overall, the results support most of the reported substrate selectivities for the catalytic domains of the JmjC KDMs.^15,16,18,19,24-29^ It is important to note that in cases of apparent discrepancies with the literature, these may reflect the use of different constructs and/or conditions and, in some cases, may arise from the omission of non-catalytic domains that would otherwise alter substrate selectivity (e.g., a TPR domain in KDM6A increases activity toward H3K27me1).^29^ Nonetheless, given that in some cases conflicting reports already exist in the literature, we hope that our results will provoke further substrate profiling work for KDMs *in vitro* and in cells. Within the subset of 6 ‘well-characterized’ subfamilies of JmjC KDMs, our results were supportive of the literature, except for the KDM4 subfamily. In the case of this subfamily, where we studied the catalytic domains of 5 KDM4s, we observed clear evidence for demethylation of H3K27me3/2 peptides. The full-length KDM4A was also able to catalyze demethylation at H3K27me3/me2. Interestingly, weak demethylase activity toward H3K27me3 was previously reported for recombinant mouse KDM4s.^34^ We emphasize that, based on our studies alone, we cannot propose that the observed KDM activity against H3K27me3/2 is of ‘biological relevance’. Nonetheless, given the promiscuity of some other 2OG oxygenases acting on proteins (e.g., FIH),^10,11,35^ there is clear potential for KDM4 subfamily members to act on H3K27me3/2 or indeed other presently unidentified substrates.

We did not observe any KDM activity with the JmjC oxygenases for which we have observed hydroxylase activity (i.e., the JmjC enzymes FIH, MINA53 and NO66) or JMJD5. From a physiological/evolutionary perspective, this is unsurprising because they belong to a different subfamily from the canonical JmjC KDMs (**Fig. 1A**).^21^ However, we do not rule out the possibility that the JmjC hydroxylases can act as KDMs; as indicated above, some 2OG oxygenases can be very promiscuous with regard to both their *in vitro* and *in vivo* substrates. As we have found with FIH, results with isolated proteins do not always predict cellular substrate selectivities and *vice versa*.

## Materials and methods

**Protein production** Catalytic domains of KDM2A,^36^ KDM4A-E,^33,37,38^ KDM6B,^39^ KDM7A,^39^ NO66,^31^ MINA53^31^, and FIH^40^ were produced in, and purified from *E. coli* as previously described. KDM3A and KDM5C were expressed and purified from Sf9 cells as described.^39^ JMJD5(1–416) in pET28a(+) (Novagen) was transformed into Rosetta DE3 *E. coli* and grown in 2 × TY media. Cells were induced at mid log phase (OD_600_ = 0.6) with 0.5 mM IPTG and grown for a further 18 hours at 25°C and harvested by centrifugation. The pellet was resuspended in 50 mM HEPES pH 7.5, 500 mM NaCl, 5 mM imidazole and 5% glycerol with an EDTA-free protease inhibitor tablet (Roche). Cells were lysed by sonication and the lysate clarified by high speed centrifugation. Lysate was purified by application to a HisTrap™ HP 5 mL column (GEHealthcare). The Ni-Sepharose column was washed with 20 column volumes of 50 mM HEPES pH 7.5, 500 mM NaCl, 20 mM imidazole and 5% glycerol, eluting with a gradient up to 250 mM imidazole over 10 column volumes. Fractions containing JMJD5 were concentrated and applied to a 300 mL Superdex 75 prep grade column pre-equilibrated in 50 mM HEPES pH 7.5, 500 mM NaCl, 1 mM DTT and 5% glycerol. Enzyme activity was assessed by 2OG turnover assay (**Fig. S13**).

**2OG turnover radioactivity assay** Recombinant JMJD5 was tested for ability to decarboxylate 1-[^14^C]-labeled 2OG, as described for other 2OG oxygenases.^13^ Reactions (100 μL) contained 4 mM sodium ascorbate, 0.288 mM 2OG, 0.0036 mM [^14^C]-2OG, 0.005 mM Fe(II), 1mM DTT, 2% catalase, 40 μM JMJD5, (10 μM inhibitor) in 50 mM HEPES pH 7.5. [^14^C]-CO_2_ radioactivity was recorded using a Beckman LS4500 liquid scintillation counter. Data shown (**Fig. 2F**) are mean values from one assay performed in triplicate. Error bars give standard error of mean.

**Peptide Synthesis** 21-mer H3 fragment peptides trimethylated at positions K4, K9, K27 and K36 were synthesized via fluorenylmethyloxycarbonyl (Fmoc)-mediated solid phase peptide synthesis (on MBHA resin) using a CS Bio 336X peptide synthesizer. Peptides were cleaved from the resin using 97.5% trifluoroacetic acid / 2.5% triisopropylsilane solution (3 hr incubation) before precipitation with cold diethyl ether. Peptides were purified by reverse-phase high-performance liquid chromatography using a Vydac C18 column (Solvent A = 0.1% trifluoroacetic acid in H_2_O, Solvent B = 0.1% trifluoroacetic acid in acetonitrile) to >95% purity as determined by MS.

**MALDI-TOF activity assays** Demethylation activities on peptides were measured by Matrix-assisted laser-desorption/ionization (MALDI) time-of-flight (TOF) mass spectrometry (MS), using a Waters Micromass MALDI micro MX^TM^ mass spectrometer in negative ion, reflectron mode. Reaction conditions varied between enzymes as optimized for each reaction (**Table S2**). Concentrations of 2OG used were maintained above the *K_m_* values for each enzyme. All reactions used 1 μM enzyme, 10 μM peptide, 2-oxoglutarate (2OG), FeSO_4_.7H_2_O and sodium ascorbate. Reactions were incubated for 60–120 min at 37°C, and were quenched with 1:2 MeOH by volume. An aliquot of the quenched reaction (1 μL) was mixed with 1 μL α-cyano-4-hydroxycinnamic acid (CHCA) matrix (Sigma) and spotted on a MALDI-TOF plate for analysis.

**MALDI competition assays** Reaction conditions for competition assays were as for MALDI-TOF activity assays, but included 2 histone peptides (10 μM each). Reactions were initiated by mixing the samples before incubation at room temperature. At each time point, 5 μL of the reaction mixture was removed, quenched with a 1:2 ratio of sample to MeOH and snap frozen in liquid nitrogen. The zero time point was quenched prior to addition of enzyme.

**Formaldehyde Dehydrogenase (FDH) Assays** Production of formaldehyde as a by-product of demethylation was observed using the FDH/NAD^+^ coupled assay.^41^ Assays contained buffer (50 mM HEPES pH 7.5, 0.01% Tween20), Fe(II) ammonium sulfate (10 μM), sodium ascorbate (100 μM), 2OG (200 μM), NAD^+^ (500 μM), enzyme (0.2–2 μM) with FDH enzyme (0.025 U, Sigma) per assay (total volume 25 μL) and were conducted at room temperature in clear-bottom black 384-well plate (Greiner). Reactions were monitored over 30 min using a PHERAstar FS (BMG Labtech) plate reader with 355 nm excitation and 460 nm emission. The *K_m_* and *V_max_* values were calculated from the reaction rate during the linear phase of formaldehyde production, as determined by the slope of the fluorescence signal recorded.

**Cell culture** HEK 293T cells were cultured at 37°C with 5% CO_2_ in DMEM (Sigma) supplemented with 10% fetal bovine serum (FBS, Invitrogen) and 1% Glutamax (Invitrogen). Full length KDM4A with FLAGx3 tag in pCDNA3 (kindly provided by Yi Zhang^42^) was transiently transfected into HEK293T using Fugene (50 μg DNA, 90 uL PEI per 15 cm diameter plate, 80% confluency, PAA Laboratories) and harvested after 24 hr. Cells were washed in PBS, lysed in 50 mM HEPES, 150 mM NaCl, 0.01% NP40, 1% protease inhibitor cocktail (SIGMA), DNAse (10 μg/ml) and incubated with 60 μL FLAG-Magnetic Beads (SIGMA) for 4 hr at 4°C. Beads were washed 4 times with lysis buffer (600 μL), 2 times with 50 mM HEPES (300 μL) to remove NP40 and NaCl before MS analysis. Beads were re-suspended in an initial volume of 60 μL for use in further assays. Demethylation reactions were conducted with 1 μL of the immobilized anti-FLAG beads with peptide (10 μM), 2OG (200 μM), FeSO_4_.7H_2_O (10 μM), sodium ascorbate (100 μM). Reactions were incubated for 120 min at 37°C and quenched with 1:2 MeOH by volume. The demethylation products were observed by MALDI.

## Supplementary Material

SI.pdf
