# Supplementary material for: Studies on the catalytic domains of multiple JmjC oxygenases using peptide substrates
Source: Epigenetics. 2015 Jan 27;9(12):1596–603. doi: 10.4161/15592294.2014.983381 (PMC4623018; doi:10.4161/15592294.2014.983381)
Supplement: SI.pdf [file kepi-09-12-983381-s001.pdf]

Supporting Information

Studies on the Catalytic Domains of Multiple JmjC Oxygenases Using Peptide Substrates

Ms Sophie T. Williams[+,1] Dr Louise J. Walport[+,1] Dr Richard J. Hopkinson[+,1] Miss Sarah K. Madden,[1] Dr Rasheduzzaman Chowdhury,[1] Prof. Christopher J. Schofield FRS[1] and Dr Akane Kawamura[1,2] *

[1] Chemistry Research Laboratory, 12 Mansfield Road, Oxford, OX1 3TA, UK.

[2] Radcliffe Department of Medicine, Division of Cardiovascular Medicine, Wellcome Trust Centre for Human Genetics, Roosevelt Drive, Oxford, OX3 7BN, UK.

[+] These authors have contributed equally to the work

* Correspondence to Dr Akane Kawamura, email: akane.kawamura@chem.ox.ac.uk.

Figures and Tables.

Note: negative data is not shown for all methylation marks for all enzymes, but is included for enzymes where there have been discrepancies in the literature.

Table S1. Peptide sequences used in MALDI and FDH activity assays. Residues which are enzymatically oxidised (hydroxylated or demethylated) are shown in bold. All peptides were purchased from Alta Bioscience or synthesised in house on a CSBio 336X solid phase peptide synthesiser. Ahx is aminohexanoic acid. All Alta Bioscience peptides have a *C*-terminal amide. Small differences in the masses of peptides are observed in the MALDI-TOF spectra due to calibration. Oxidation (+16 Da) of some biotinylated peptides was observed in the mass spectra in some instances.

Histone Mark / Protein	Amino Acid Sequence	Average Mass / Da	Source
H3K4	Biotin-Ahx-ART**Kme1**QTARKSTGGKAPRKQLA	2607.5	Alta Bioscience
H3K4	Biotin- Ahx -ART**Kme2**QTARKSTGGKAPRKQLA	2621.5	Alta Bioscience
H3K4	Biotin- Ahx -ART**Kme3**QTARKSTGGKAPRKQLA	2635.5	Alta Bioscience
H3K9	Biotin- Ahx -ARTKQTAR**Kme1**STGGKAPRKQLA	2607.5	Alta Bioscience
H3K9	Biotin- Ahx -ARTKQTAR**Kme2**STGGKAPRKQLA	2621.5	Alta Bioscience
H3K9	Biotin- Ahx -ARTKQTAR**Kme3**STGGKAPRKQLA	2635.5	Alta Bioscience
H3K27	Biotin- Ahx -KAPRKQLATKAAR**Kme1**SAPATGG	2461.3	Alta Bioscience
H3K27	Biotin- Ahx -KAPRKQLATKAAR**Kme2**SAPATGG	2475.3	Alta Bioscience
H3K27	Biotin- Ahx -KAPRKQLATKAAR**Kme3**SAPATGG	2489.3	Alta Bioscience
H3K36	Biotin- Ahx -SAPATGGV**Kme1**KPHRYRPGTVAL	2516.4	Alta Bioscience
H3K36	Biotin- Ahx -SAPATGGV**Kme2**KPHRYRPGTVAL	2531.4	Alta Bioscience
H3K36	Biotin- Ahx -SAPATGGV**Kme3**KPHRYRPGTVAL	2545.4	Alta Bioscience
H3K4	ART**Kme3**QTARKSTGGKAPRKQLA	2297.6	In house
H3K9	ARTKQTAR**Kme3**STGGKAPRKQLA	2297.6	In house
H3K27	KAPRKQLATKAAR**Kme3**SAPATGG	2151.1	In house
H3K36	SAPATGGV**Kme3**KPHRYRPGTVAL	2206.5	In house
Rpl27a	GRGNAGGL**H**HHRINFDKYHP	2281.1	In house
Rpl8	NPVEHPFGGG**N**HQHIGKPST	2110.3	In house
Synthetic ankyrin	HLEVVKLLLEAGADV**N**AQDK	2161.2	In house

Table S2. Demethylation assay conditions.

**Enzyme**	**[2OG] / µM**	**[Ascorbate] / µM**	**[Fe^{II}] / µM**	**Buffer Conditions**
KDM2A	200	100	10	50 mM HEPES pH 7.5
KDM3A	200	100	10	50 mM HEPES pH 7.5, 150 mM NaCl
KDM4A-E	200	100	10	50 mM HEPES pH 7.5
KDM5C	200	100	10	50 mM HEPES pH 7.5
KDM6A	200	100	10	50 mM HEPES pH 7.5, 150 mM NaCl, 5% glycerol
KDM6B	200	100	10	50 mM HEPES pH 7.5, 150 mM NaCl, 5% glycerol
KDM7A	200	100	10	50 mM HEPES pH 7.5
JMJD5	200	100	10	50 mM HEPES pH 7.5, 150 mM NaCl, 5% glycerol, 1 mM DTT
MINA53	200	100	10	50 mM HEPES pH 7.5, 150 mM NaCl, 5% glycerol, 1 mM DTT
NO66	200	100	10	50 mM HEPES pH 7.5, 150 mM NaCl, 5% glycerol, 1 mM DTT
FIH	200	100	10	50 mM Tris pH 7.5, 150 mM NaCl

Table S3. Crystallographic data processing and refinement statistics. Sample composition of 1(KDM4A):5(NOG):peptide(10) was used, with KDM4A[1] at 10mg/ml.

**Measurement**	**KDM4A + 20 mer H3K27me3 Peptide PDB ID: 4V2W**	**KDM4A + 5 mer H3K27me3 Peptide PDB ID: 4V2V**
**Crystallization and cryo-protection**		
Crystallization conditions	0.02 M sodium/potassium phosphate, 0.1 M Bis Tris propane pH 7.5, 20 % w/v PEG 3350	0.2 M ammonium chloride, 20 % w/v PEG 3350
Vapour diffusion conditions	Sitting drop (300 nl), protein-to-well ratio, 2:1, 277K	Sitting drop (300 nl), protein-to-well ratio, 1:1, 277K
Cryo-protection (% supplemented with well condition)	25% glycerol	25% glycerol
**Data Collection**		
Data processing	MOSFLM[2], SCALA[3]	MOSFLM[2], SCALA[3]
Space Group	$P2_12_12$	$P2_12_12$
Cell dimensions a,b,c (Å)	100.66	101.02
	149.73	149.91
	57.50	57.38
Resolution (Å)	60.07 – 1.81 (1.91 – 1.81)*	53.59 – 2.00 (2.11 – 2.00)*
No. of unique reflections	79999 (11459)*	59803 (8616)*
Completeness (%)	99.9 (99.3)*	100 (100)*
Redundancy	6.9 (6.2)*	5.8 (5.5)*
R_{sym}**	0.098 (0.889)*	0.088 (0.786)*
Mean $I/\sigma(I)$	9.9 (2.0)*	11.0 (2.2)*
**Refinement**	PHENIX[4]	PHENIX[4]
R_{factor}	0.1724	0.1860
R_{free}	0.2082	0.2208
R.m.s. deviation		
Bond length, Å	0.01	0.008
Bond angle, °	1.32	1.18

*Highest resolution shell shown in parenthesis.

**$R_{sym} = \sum|I - \langle I \rangle|/\sum I$, where I is the intensity of an individual measurement and $\langle I \rangle$ is the average intensity from multiple observations.

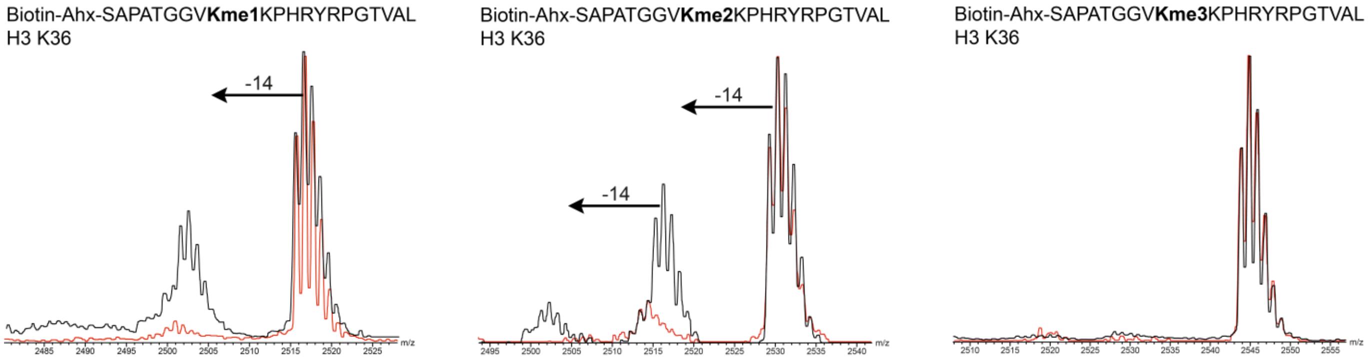

Figure S1. Representative MALDI MS showing KDM2A-catalysed demethylation of H3 fragment peptides methylated at K36. Peptide only assay (red) overlaid with enzyme reaction (black).

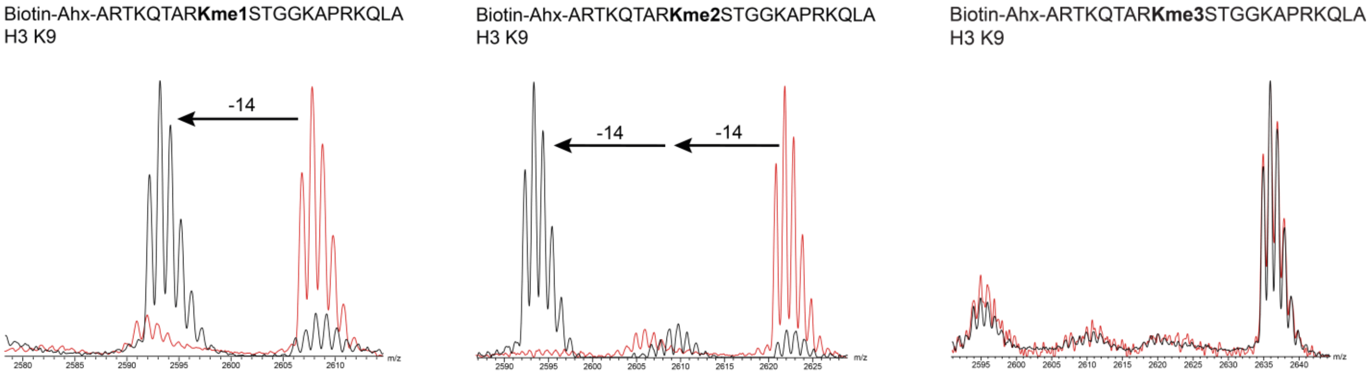

Figure S2. Representative MALDI MS showing KDM3A-catalysed demethylation of H3 fragment peptides methylated at K9. Peptide only assay (red) overlaid with enzyme reaction (black).

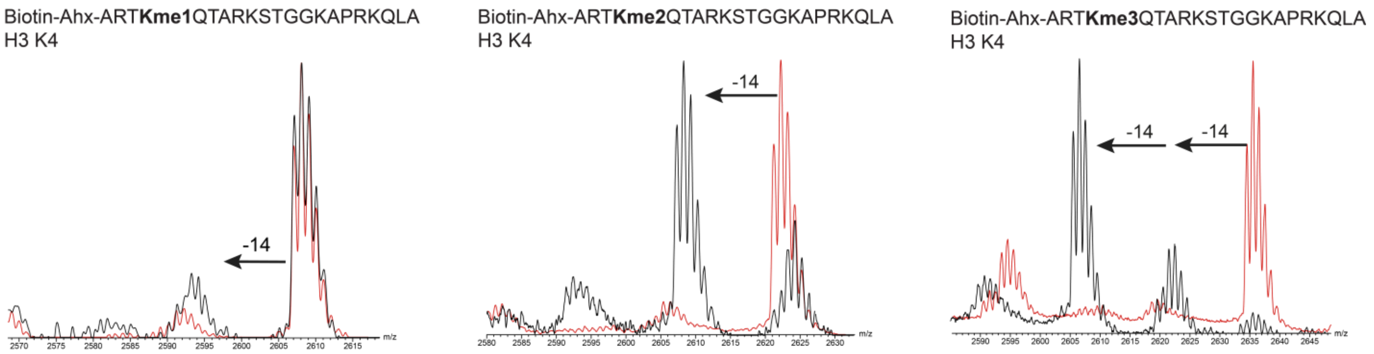

Figure S3. Representative MALDI MS showing KDM5C-catalysed demethylation of H3 fragment peptides methylated at K4. Peptide only assay (red) overlaid with enzyme reaction (black).

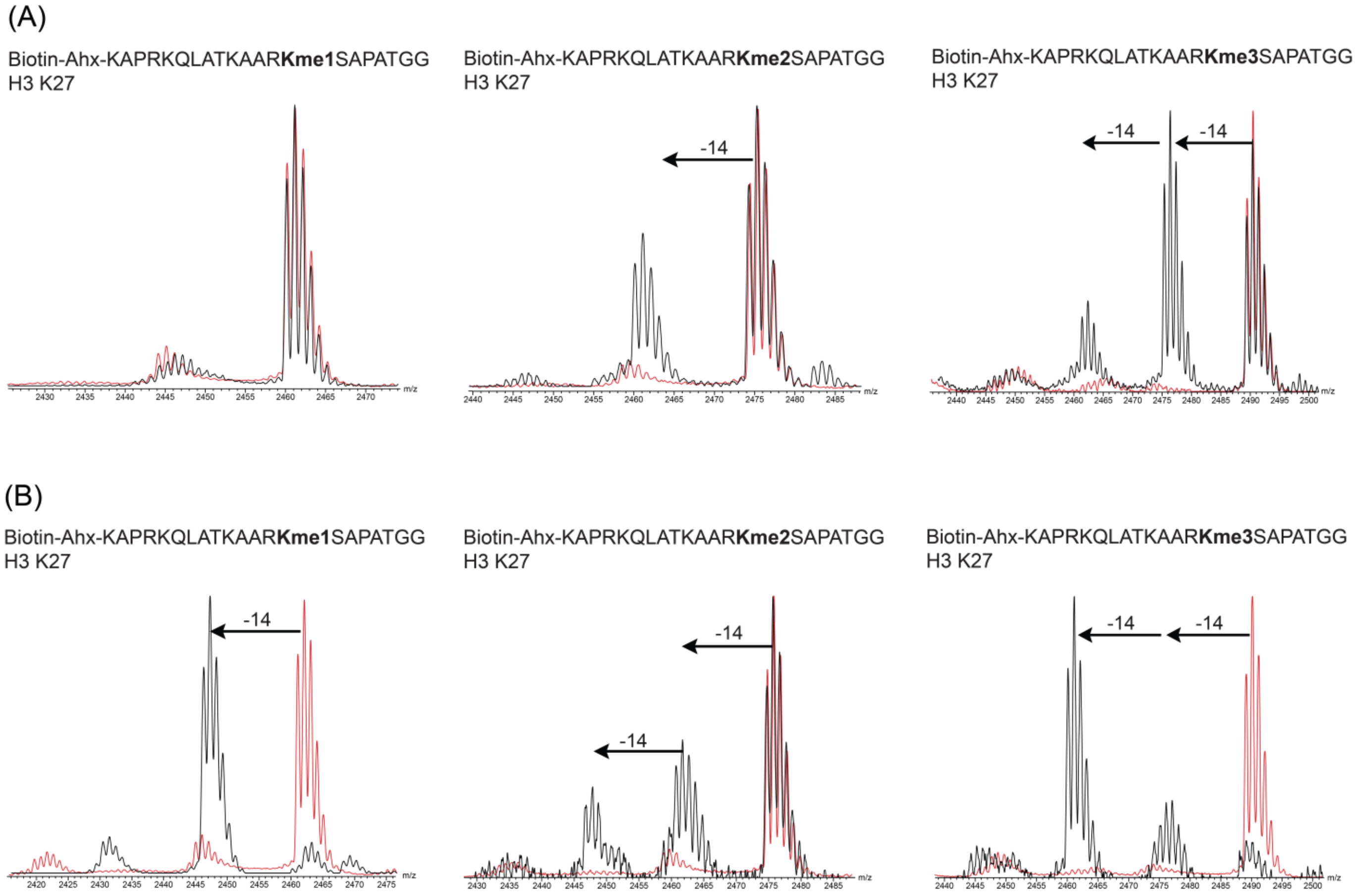

Figure S4. Representative MALDI MS showing KDM6A (A) and B (B) catalysed demethylation of H3 fragment peptides methylated at K27. Peptide only assay (red) overlaid with enzyme reaction (black).

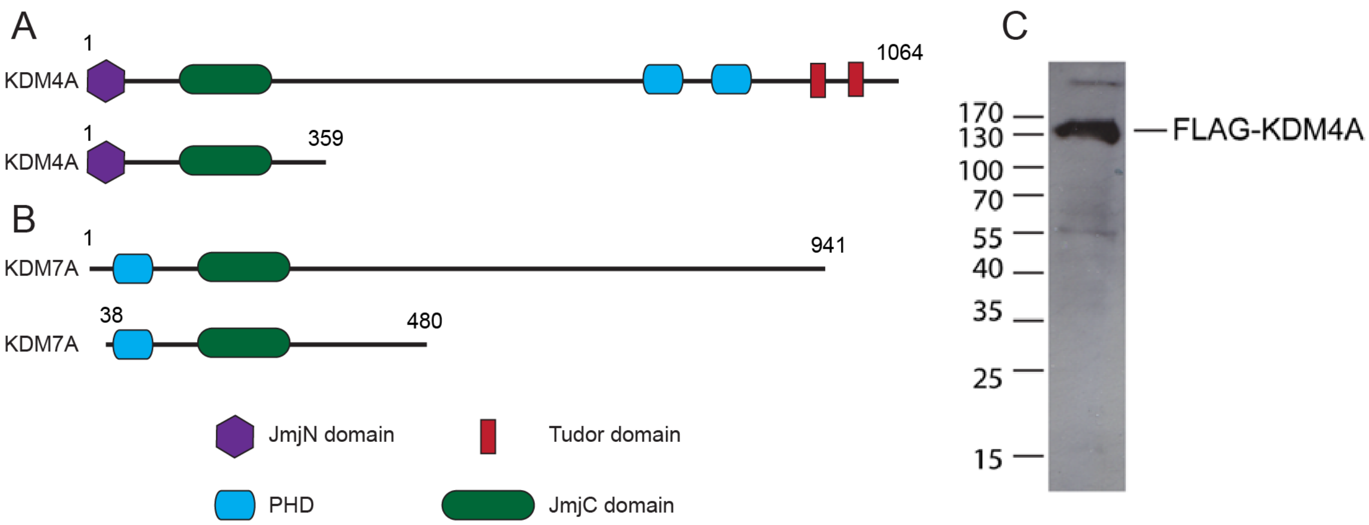

Figure S5. Domain organisation of (A) KDM4A and (B) KDM7A with (C) Western-Blot analysis of FLAG-KDM4A$_{1\text{-}1064}$. In figures A and B, the upper figure shows the full length protein domain structure (as used for KDM4A), and below shows truncated domain structure in the constructs used for *in vitro* studies (as used for KDM4A and KDM7A). Figure C shows a Western-Blot of immunoprecipitated FLAG-KDM4A 1-1064 purified from HEK293T probed using anti-FLAG antibody. Predicted 3xFLAG-KDM4A weight is 123.4 kDa.

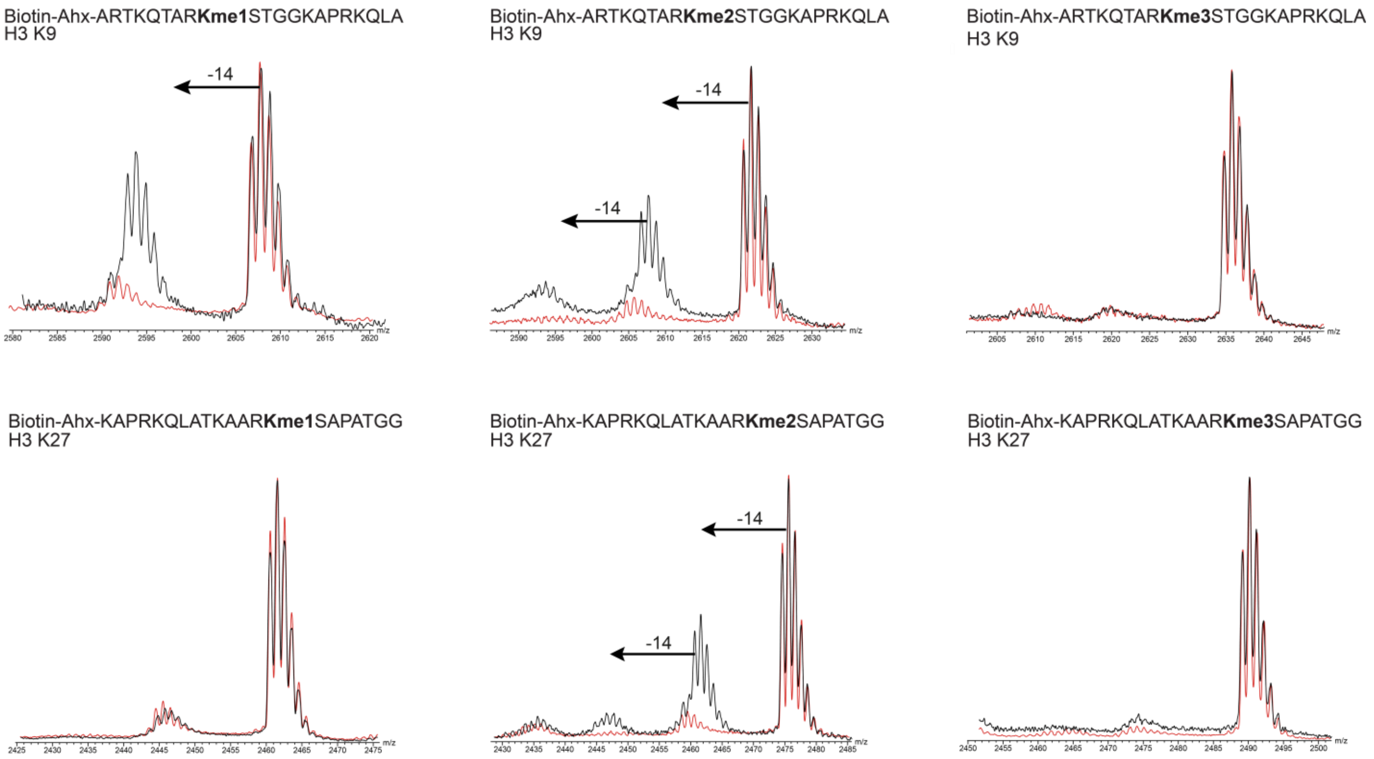

Figure S6. Representative MALDI MS showing KDM7A catalysed demethylation of H3 fragment peptides methylated at K9 and K27.[5] Peptide only assay (red) overlaid with enzyme reaction (black).

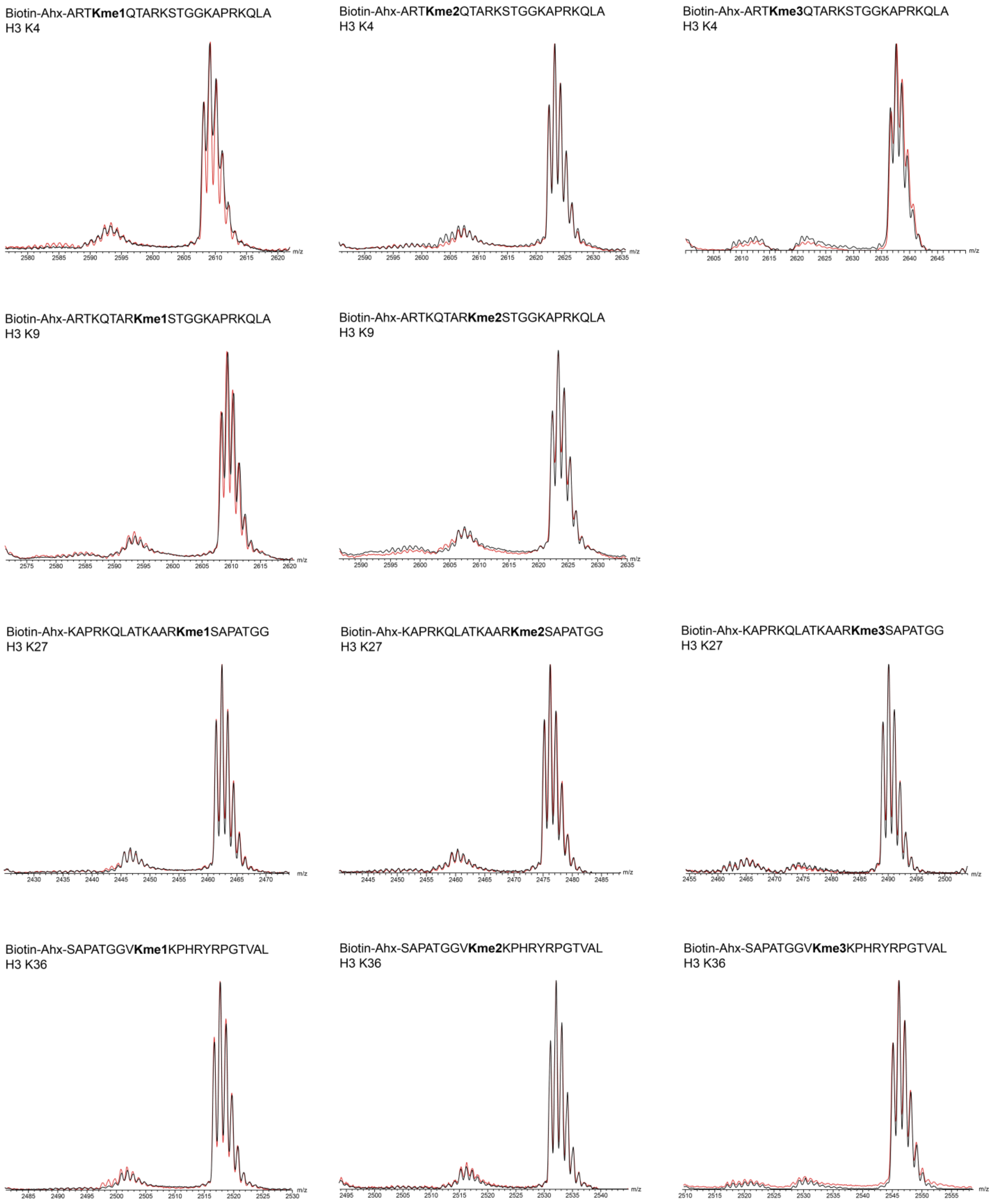

Figure S7. Representative MALDI MS showing the absence of demethylation of histone peptides by MINA53.[6] Peptide only assay (red) overlaid with enzyme reaction (black).

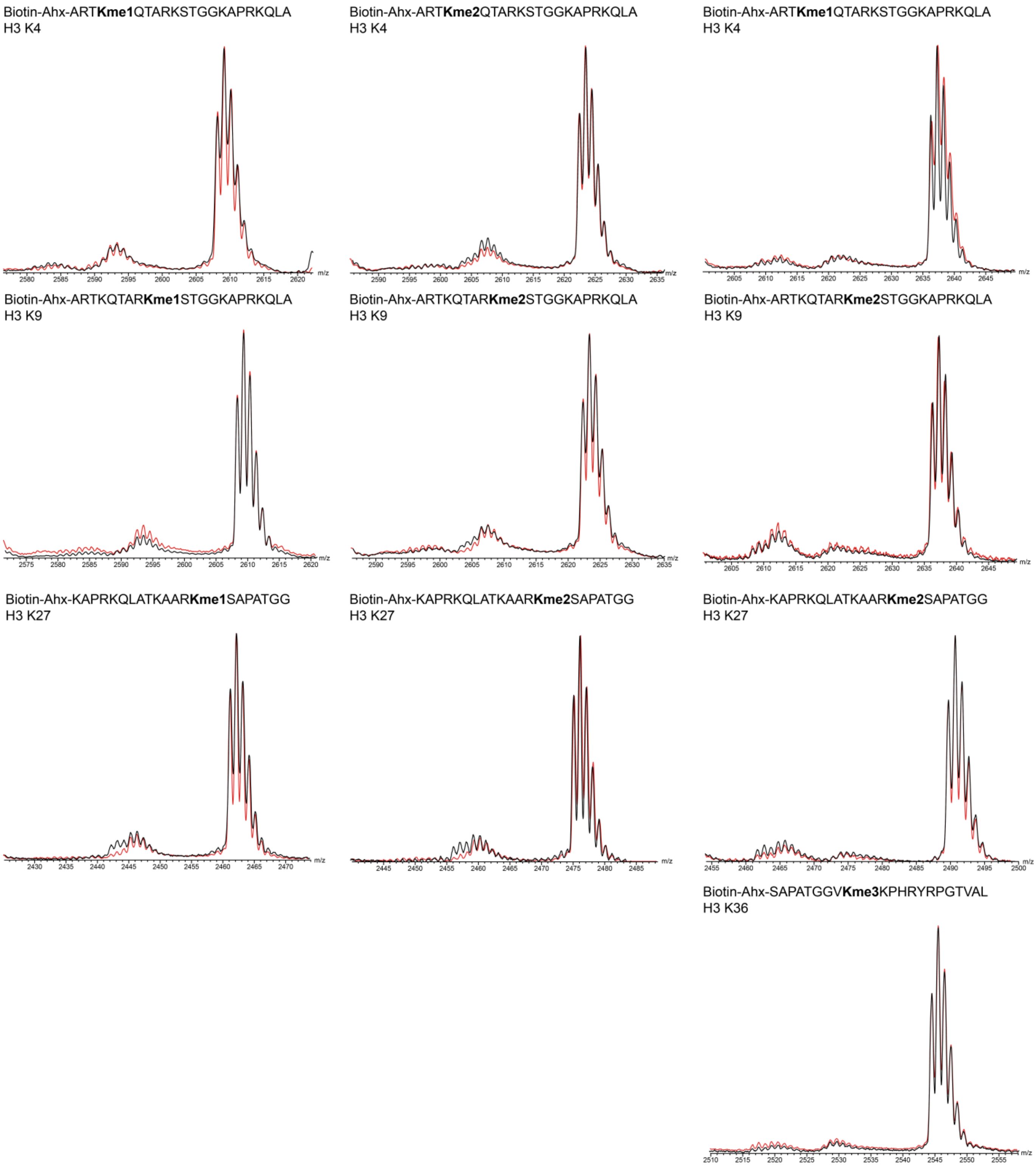

Figure S8. Representative MALDI MS showing the absence of demethylation of histone peptides by NO66.[6]

Peptide only assay (red) overlaid with enzyme reaction (black).

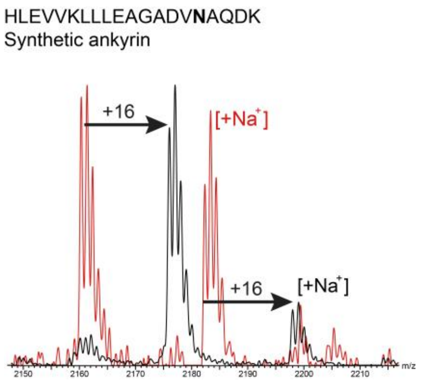

Figure S9. Hydroxylation of synthetic ankyrin peptide by FIH.[7] Peptide only assay (red) shown with enzyme reaction (black).

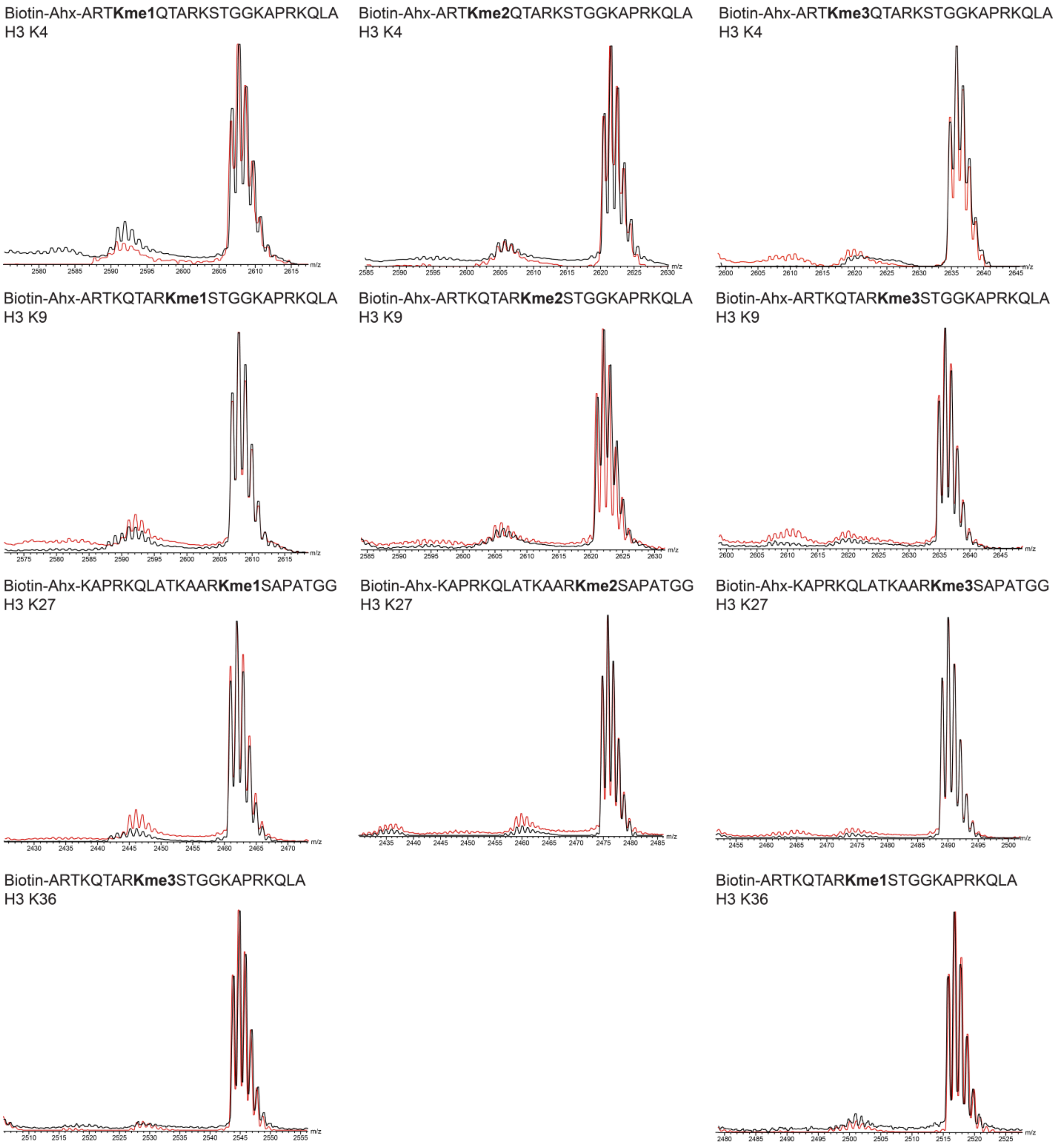

Figure S10. Representative MALDI MS showing lack of demethylation of histone peptides by JMJD5.

Peptide only assay (red) shown with enzyme reaction (black).

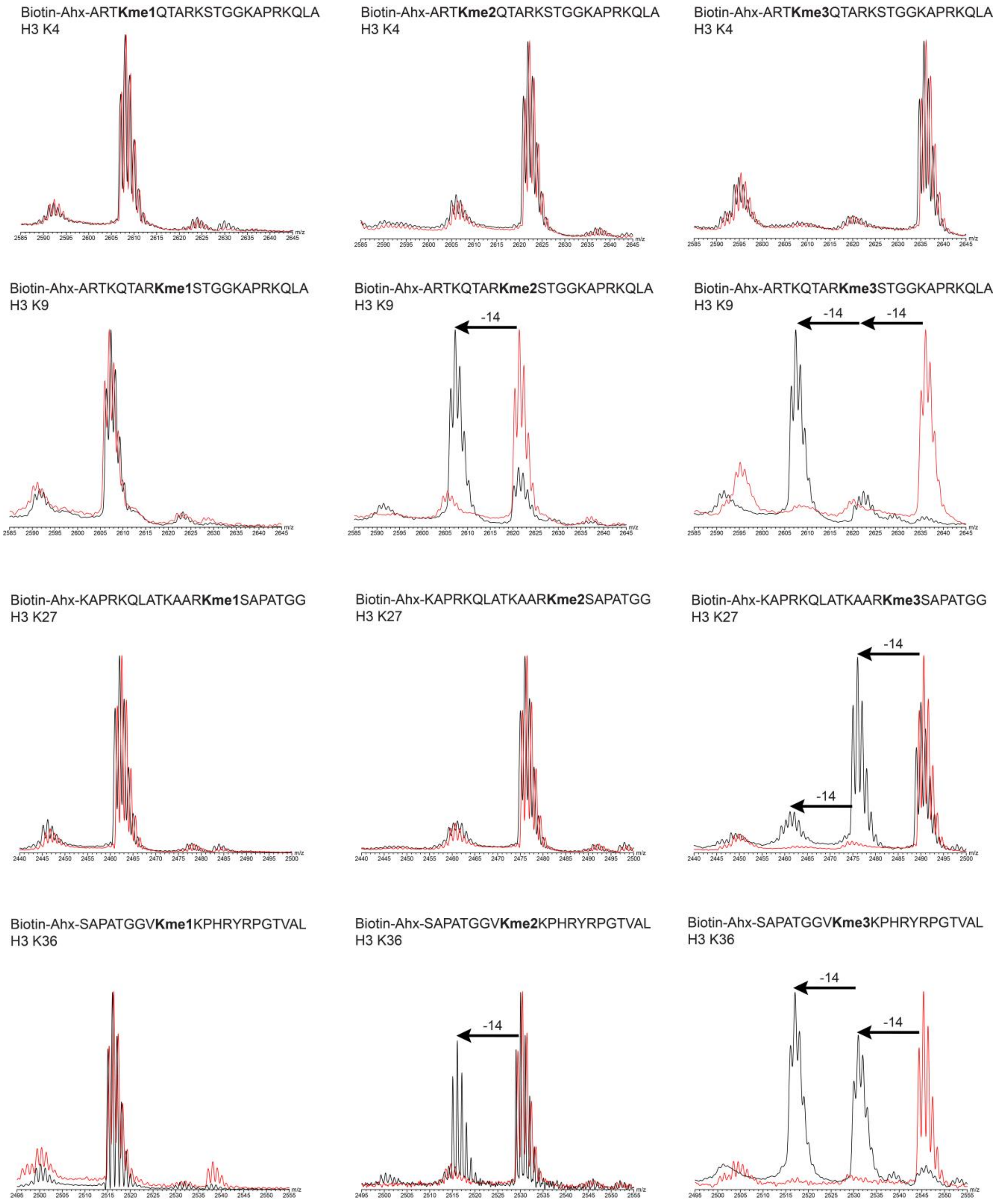

Figure S11. Representative MALDI MS showing KDM4A-catalysed demethylation of H3 fragment peptides methylated at K4, K9, K27 and K36. Peptide only assay (red) overlaid with enzyme reaction (black).

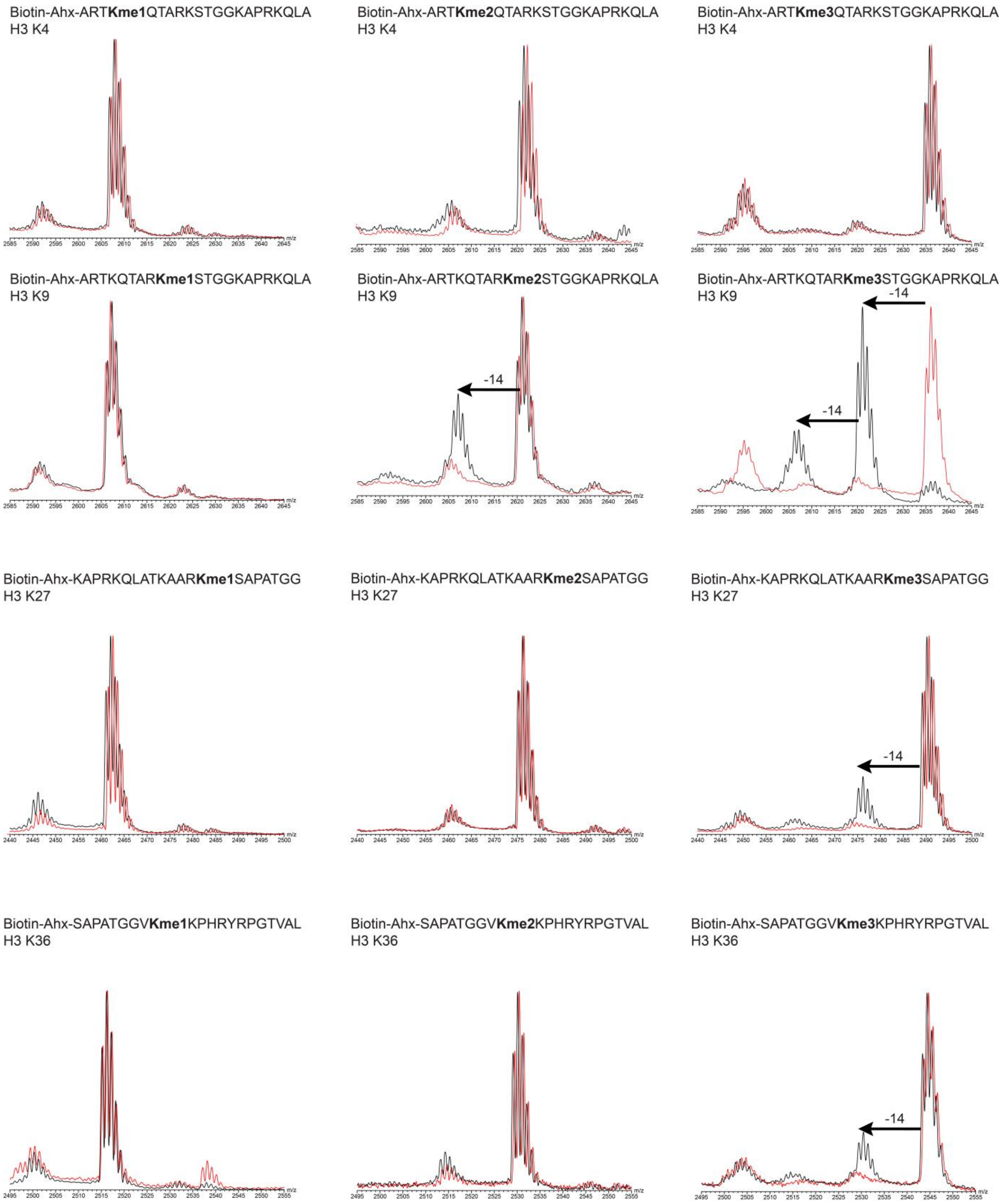

Figure S12. Representative MALDI MS showing KDM4B-catalysed demethylation of H3 fragment peptides methylated t K9, K27 and K36. Peptide only assay (red) overlaid with enzyme reaction (black).

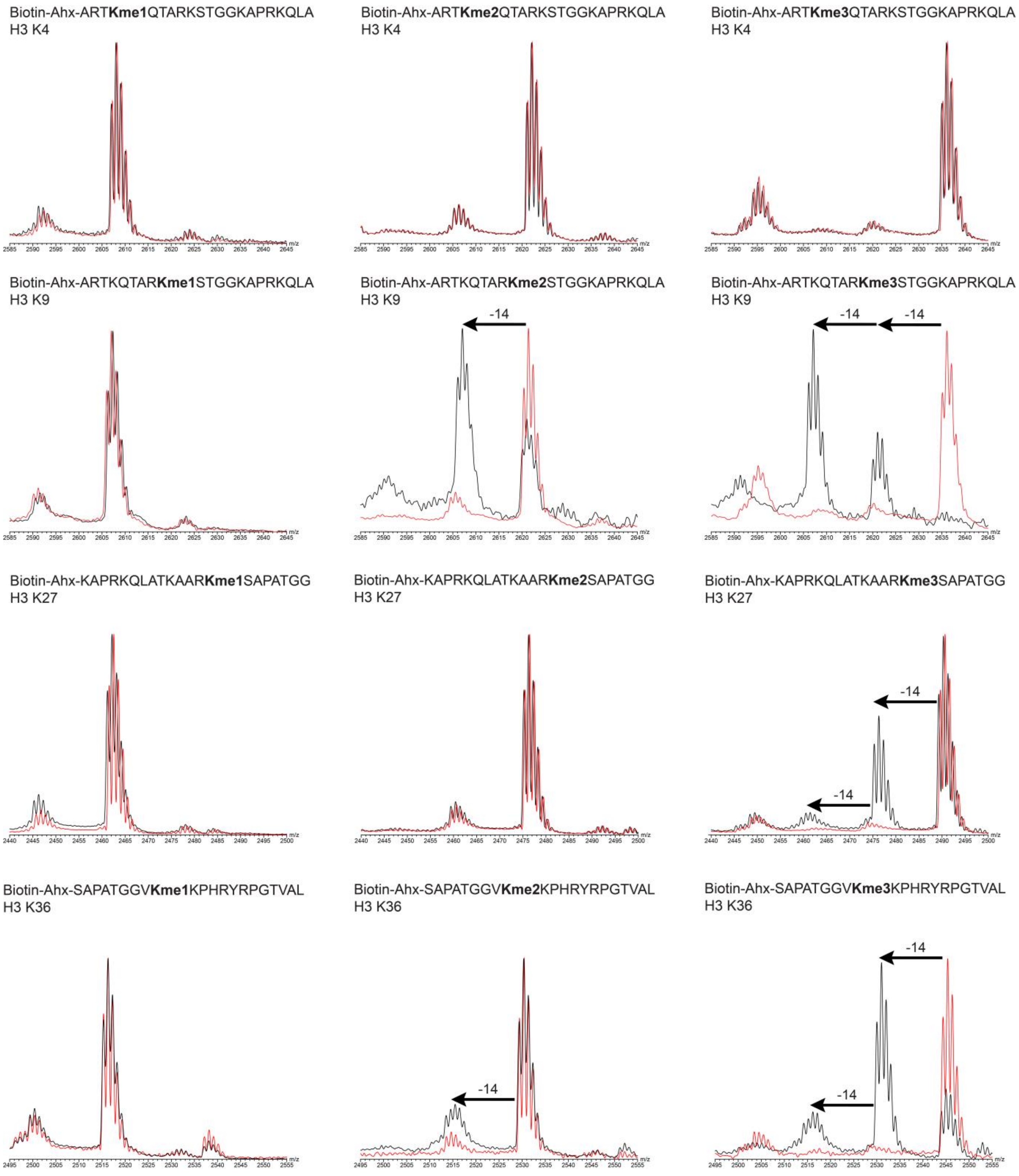

Figure S13. Representative MALDI MS showing KDM4C-catalysed demethylation of H3 fragment peptides methylated at K9, K27 and K3. Peptide only assay (red) overlaid with enzyme reaction (black).

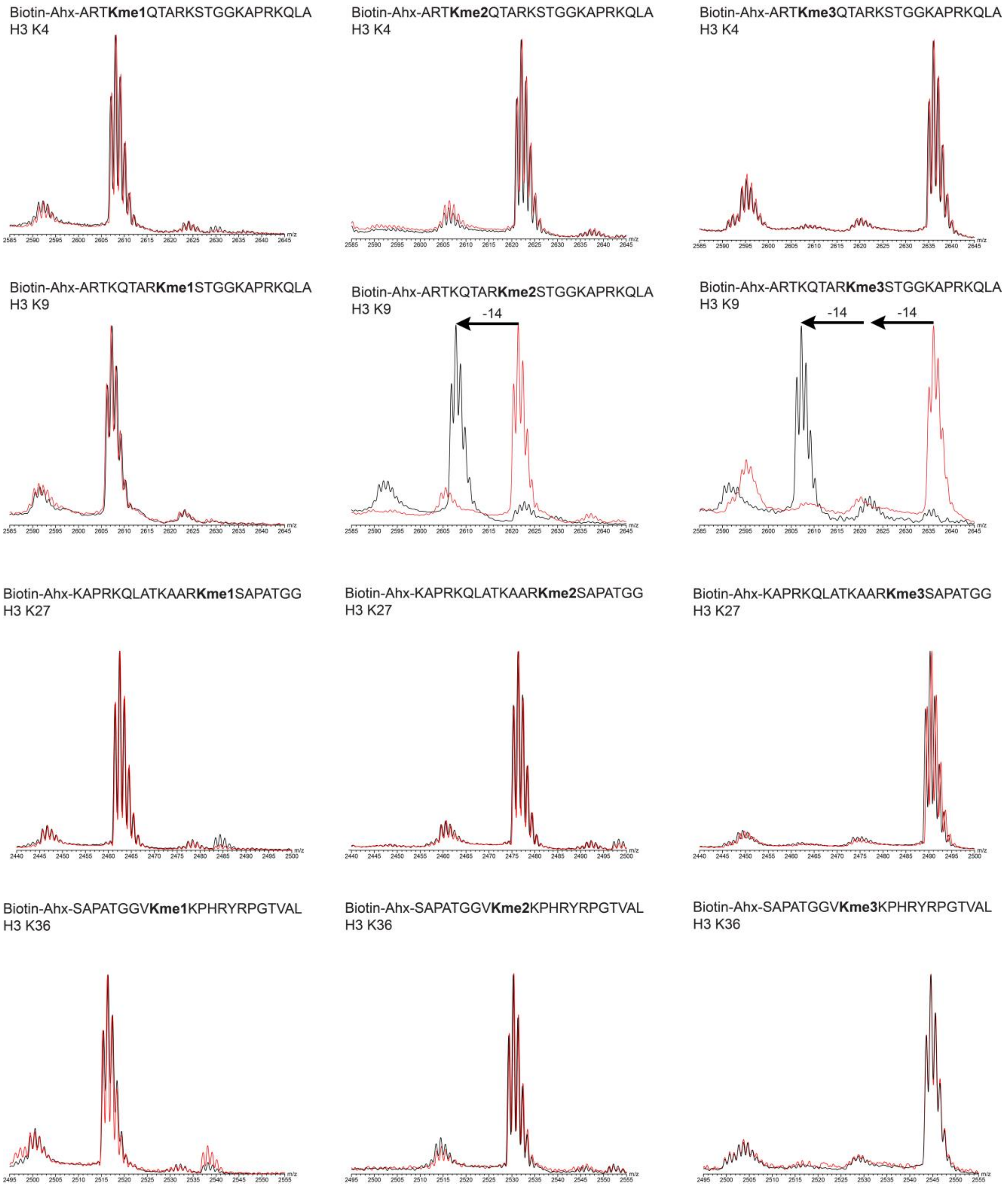

Figure S14. Representative MALDI MS showing KDM4D-catalysed demethylation of H3 fragment peptides methylated at K9 and K27. Peptide only assay (red) overlaid with enzyme reaction (black).

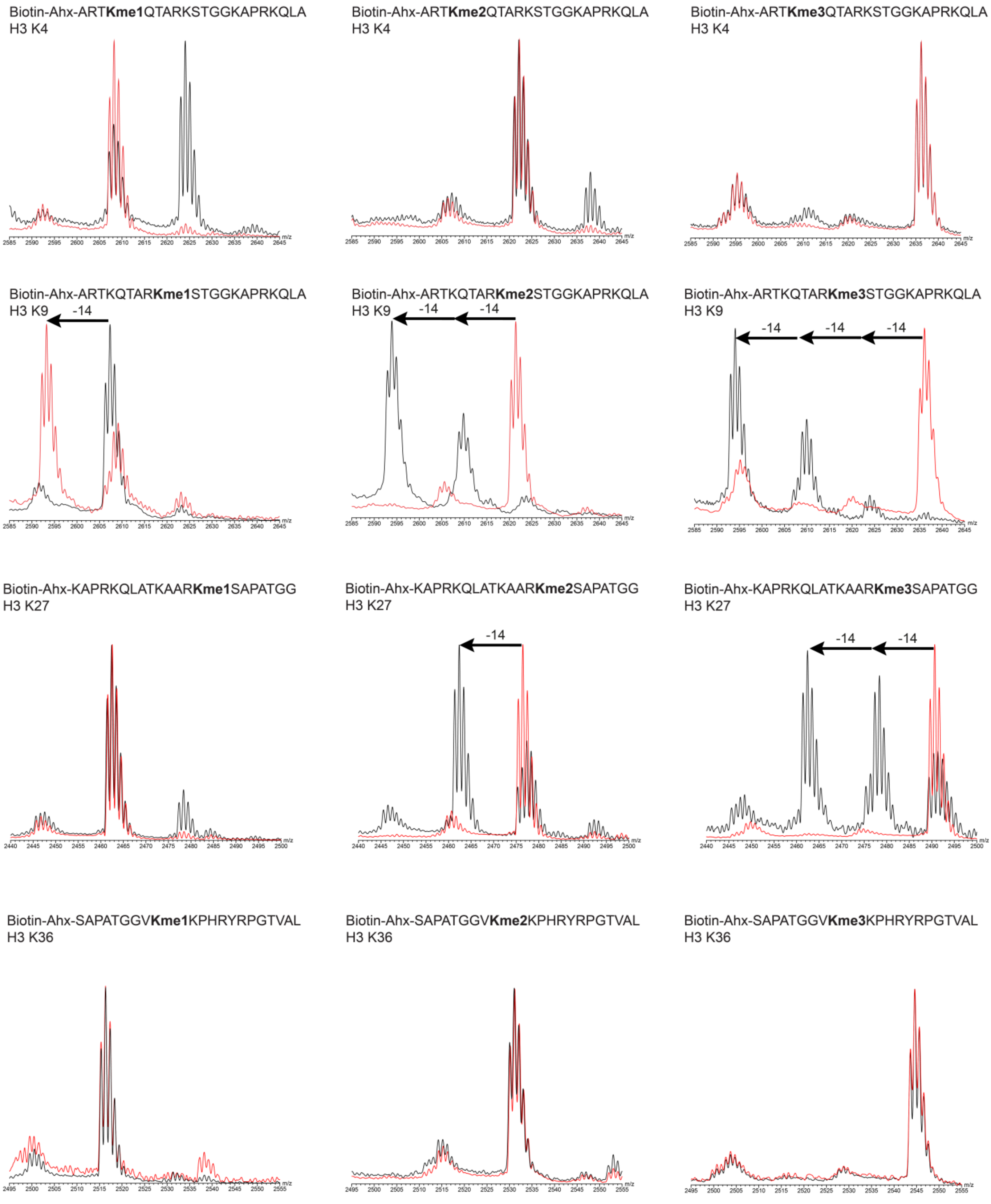

Figure S15. Representative MALDI MS showing KDM4E-catalysed demethylation of H3 fragment peptides methylated at K9 and K27. Peptide only assay (red) overlaid with enzyme reaction (black).

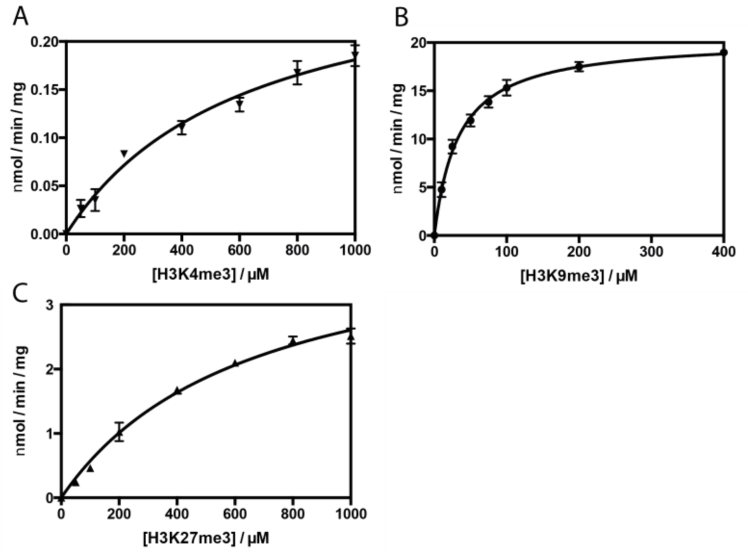

Figure S16. Michaelis-Menten curves for KDM4A with histone H3 trimethylated peptides. Initial rates over a range of peptide concentrations were determined using the FDH assay with saturating 2OG concentrations of 200 μM.

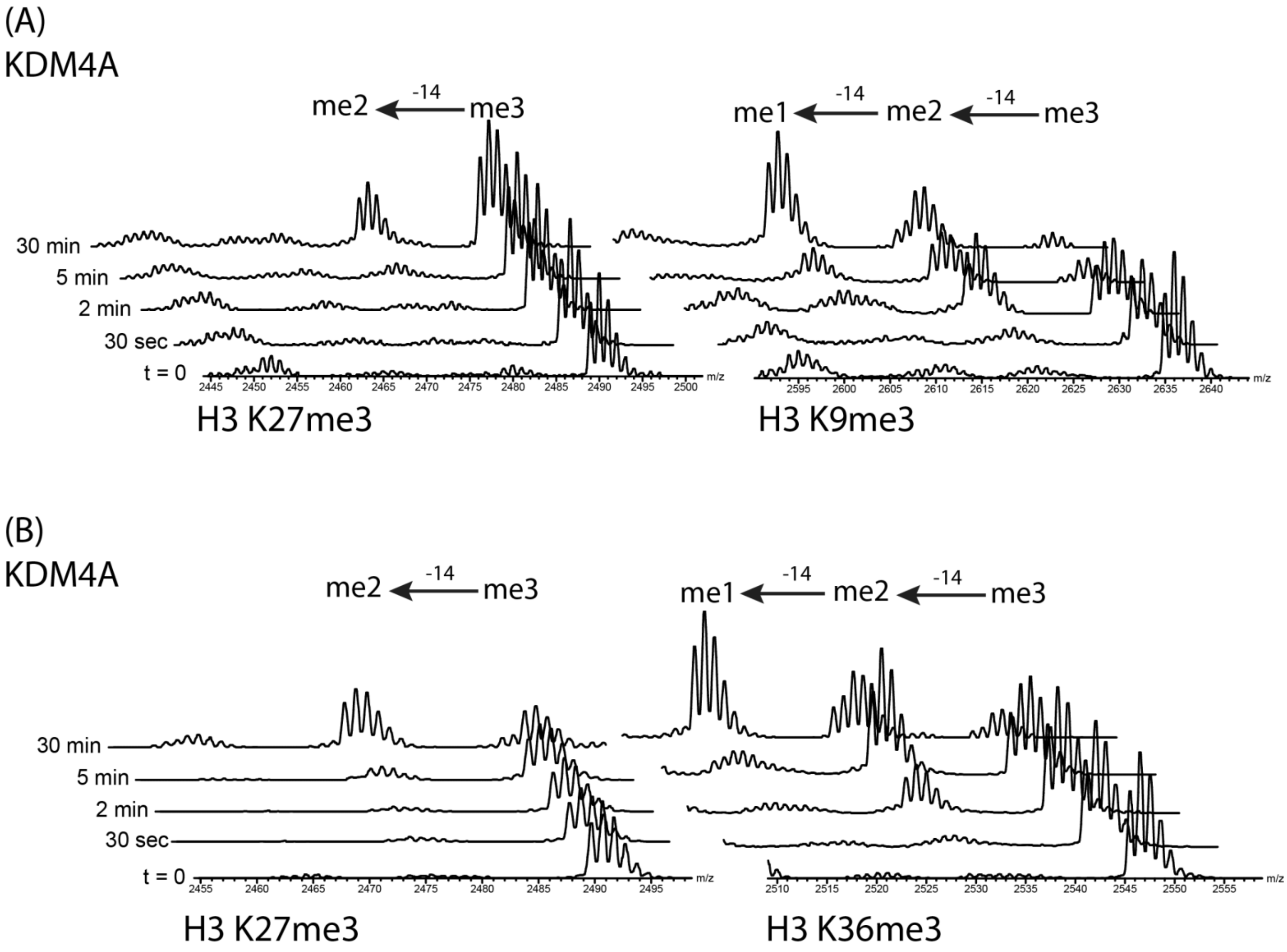

Figure S17. Competition for demethylation by KDM4A between H3 K27me3 and (A) K9me3 or (B) K36me3 in a 1:1 concentration ratio, as analysed by MALDI MS.

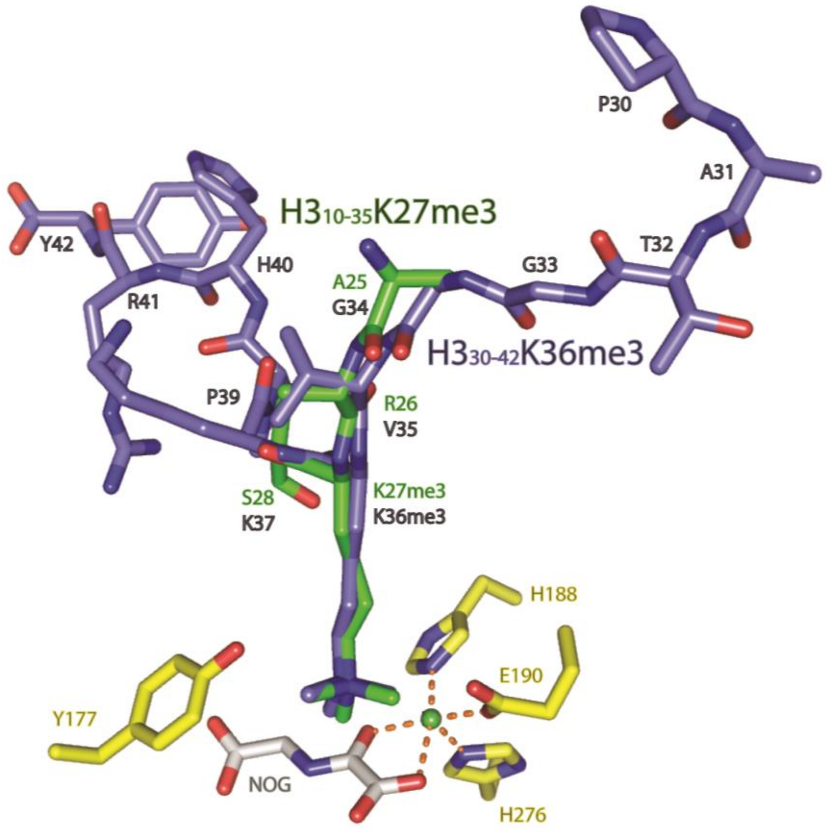

Figure S18. View from an X-ray crystal structure of the catalytic domain of KDM4A in complex with an H3K27me3 fragment peptide overlaid with $H3_{30\text{-}42}$K36me3 (PDB ID: 2YBS). Nickel (Ni, green) and N-oxalylglycine (NOG, grey) substitute for iron (II) and 2OG, respectively. Active site residues from PDB 4V2W are shown in yellow (Tyr177, His188, Glu190, His276, Asp290). The position of the K27me3 residue of the fragment peptide correlates closely with that reported for H3K36me3, although the surrounding peptide sequence differs significantly (see peptide sequences in Figure 4).

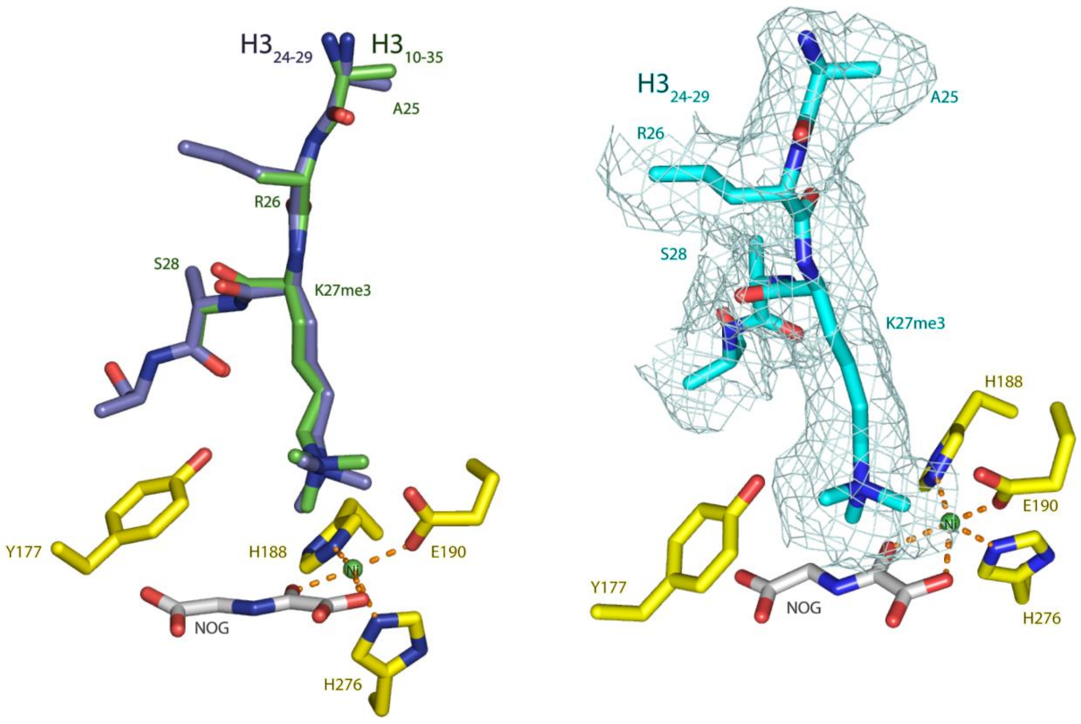

Figure S19. View from an X-ray crystal structure of KDM4A complexed with a shorter 5 residue $H3_{10-35}$ K27me3 peptide (purple/blue). The $H3_{24-29}$K27 5 residue peptide (purple) is shown overlaid with the $H3_{10-35}$K27 25 residue peptide (green). Nickel (Ni, green) and N-oxalylglycine (NOG, grey) substitute for iron (II) and 2OG, respectively. Active site residues from PDB 4V2V are shown in yellow (Tyr177, His188, Glu190, His276, Asp290).

References

1. Ng SS, Kavanagh KL, McDonough MA, Butler D, Pilka ES, Lienard BM, Bray JE, Savitsky P, Gileadi O, von Delft F, et al. Crystal structures of histone demethylase JMJD2A reveal basis for substrate specificity. Nature 2007; 448:87-91.
2. Battye TG, Kontogiannis L, Johnson O, Powell HR, Leslie AG. iMOSFLM: a new graphical interface for diffraction-image processing with MOSFLM. Acta crystallographica Section D, Biological crystallography 2011; 67:271-81.
3. Winn MD, Ballard CC, Cowtan KD, Dodson EJ, Emsley P, Evans PR, Keegan RM, Krissinel EB, Leslie AG, McCoy A, et al. Overview of the CCP4 suite and current developments. Acta crystallographica Section D, Biological crystallography 2011; 67:235-42.
4. Adams PD, Afonine PV, Bunkoczi G, Chen VB, Davis IW, Echols N, Headd JJ, Hung LW, Kapral GJ, Grosse-Kunstleve RW, et al. PHENIX: a comprehensive Python-based system for macromolecular structure solution. Acta crystallographica Section D, Biological crystallography 2010; 66:213-21.
5. Horton JR, Upadhyay AK, Qi HH, Zhang X, Shi Y, Cheng X. Enzymatic and structural insights for substrate specificity of a family of jumonji histone lysine demethylases. Nature structural & molecular biology 2010; 17:38-43.
6. Ge W, Wolf A, Feng T, Ho CH, Sekirnik R, Zayer A, Granatino N, Cockman ME, Loenarz C, Loik ND, et al. Oxygenase-catalyzed ribosome hydroxylation occurs in prokaryotes and humans. Nature chemical biology 2012; 8:960-2.
7. Yang M, Ge W, Chowdhury R, Claridge TD, Kramer HB, Schmierer B, McDonough MA, Gong L, Kessler BM, Ratcliffe PJ, et al. Asparagine and aspartate hydroxylation of the cytoskeletal ankyrin family is catalyzed by factor-inhibiting hypoxia-inducible factor. The Journal of biological chemistry 2011; 286:7648-60.
